# “Future Patient” Telerehabilitation for Patients With Heart Failure: Protocol for a Randomized Controlled Trial

**DOI:** 10.2196/14517

**Published:** 2019-09-19

**Authors:** Birthe Dinesen, Lars Dittmann, Josefine Dam Gade, Cecilia Klitgaard Jørgensen, Malene Hollingdal, Soeren Leth, Camilla Melholt, Helle Spindler, Jens Refsgaard

**Affiliations:** 1 Laboratory for Welfare Technologies - Telehealth & Telerehabilitation Department of Health Science and Technology Aalborg University Aalborg Denmark; 2 Department of Photonics Engineering Danish Technical University Copenhagen Denmark; 3 Cardiology Ward Regional Hospital in Viborg Viborg Denmark; 4 Department of Psychology and Behavviroral Sciences Aarhus University Aarhus Denmark

**Keywords:** heart failure, telerehabilitation, research design, quality of life, patient education, user-driven innovation

## Abstract

**Background:**

Cardiovascular disease is the leading cause of mortality worldwide, accounting for 13%-15% of all deaths. Cardiac rehabilitation has poor compliance and adherence. Telerehabilitation has been introduced to increase patients’ participation, access, and adherence with the help of digital technologies. The target group is patients with heart failure. A telerehabilitation program called “Future Patient” has been developed and consists of three phases: (1) titration of medicine (0-3 months), (2) implementation of the telerehabilitation protocols (3 months), and (3) follow-up with rehabilitation in everyday life (6 months). Patients in the Future Patient program measure their blood pressure, pulse, weight, number of steps taken, sleep, and respiration and answer questions online regarding their well-being. All data are transmitted and accessed in the HeartPortal by patients and health care professionals.

**Objective:**

The aim of this paper is to describe the research design, outcome measures, and data collection techniques in the clinical test of the Future Patient Telerehabilitation Program for patients with heart failure.

**Methods:**

A randomized controlled study will be performed. The intervention group will follow the Future Patient Telerehabilitation program, and the control group will follow the traditional cardiac rehabilitation program. The primary outcome is quality of life measured by the Kansas City Cardiomyopathy Questionnaire. Secondary outcomes are development of clinical data; illness perception; motivation; anxiety and depression; health and electronic health literacy; qualitative exploration of patients’, spouses’, and health care professionals’ experiences of participating in the telerehabilitation program; and a health economy evaluation of the program. Outcomes were assessed using questionnaires and through the data generated by digital technologies.

**Results:**

Data collection began in December 2016 and will be completed in October 2019. The study results will be published in peer-reviewed journals and presented at international conferences. Results from the Future Patient Telerehabilitation program are expected to be published by the spring of 2020.

**Conclusions:**

The expected outcomes are increased quality of life, increased motivation and illness perception, reduced anxiety and depressions, improved electronic health literacy, and health economics benefits. We expect the study to have a clinical impact for future telerehabilitation of patients with heart failure.

**Trial Registration:**

ClinicalTrials.gov NCT03388918; https://clinicaltrials.gov/ct2/show/NCT03388918

**International Registered Report Identifier (IRRID):**

DERR1-10.2196/14517

## Introduction

Cardiovascular disease is the leading cause of mortality [1-4], accounting for 13%-15% of all deaths worldwide and 24.8% of all deaths in Europe [3]. A majority of cardiovascular disease mortalities are caused by heart failure. Despite advances in heart failure treatment in the past decade, increased lifespan along with obesity and unhealthy lifestyle has caused a continuing increase in the prevalence of heart failure [4,5]. An increase in the prevalence combined with an increasing elderly population has increased the health care costs and the number of people with disabilities [6]. The rehabilitation of cardiac patients aims to improve patients’ recovery, functional capacity, psychosocial well-being, and quality of life by using interventions such as physical activity, improved diet, weight control, psychosocial coping, and disease management [3,7,8]. These kinds of lifestyle-based interventions are crucial for patient recovery. However, cardiac rehabilitation programs have poor compliance and adherence [3]. Patients may take their medication regularly, but they find it difficult to alter lifestyle routines. To address this problem, telerehabilitation has been introduced to increase patient participation, access, and adherence with the help of information and communication technology [3,9].

To date, telehealth and telerehabilitation in heart failure has focused on systems that use hemodynamic measurements (ie, blood pressure and pulse), respiration, weight, and subjective questions to assess the risks of worsening heart failure. The responses are sent to health care providers, who then act on these data. Generally, patients do not see their own data. Instead, they wait for a health care provider to communicate to them on whether their heart failure has worsened [10]. A review from 2014 concluded that telemonitoring of patients with heart failure helped minimize decompensation of heart failure in small, single-center use when providers and patients were dedicated or when communication was direct through structured telephone support. However, when telemonitoring was used for the care of populations (in large trials), it proved no better than conventional care [11]. When researchers used questionnaires and qualitative research designs to assess tailored telemonitoring in patients with heart failure, patients reported improved self-care abilities and self-efficacy [12-14].

Patients’ preferences and their choice of smart technology in telemonitoring have been assessed in a transatlantic multisite study [15]. Patients with heart failure (n=208) stated that they preferred mobile devices and self-tracking devices for monitoring everyday activity. The use of activity trackers in patients with heart failure had a motivational effect, helping patients increase the number of steps per day [3,16]. In some cases, monitoring daily activity can also be used as predictors of patient health. The Future Patient study [17] described here proposes a new approach in self-management of patients with heart failure by using self-tracking technologies for monitoring physical activity, sleep, respiration, and pulse at night by using low-cost, commercially available, Conformité Européene–marked trackers that can passively “observe” patients in everyday life and make data available for both patients and health care professionals. Based on participatory design [18-20], the Future Patient Telerehabilitation (FPT) program has been developed in collaboration with patients with heart failure; relatives; health care professionals from hospital and health care centers; companies; and an interdisciplinary research team comprising professionals from engineering, psychology, medicine, nursing, and organizational sociology.

The overall purpose of the FPT study is to develop a telerehabilitation program that helps increase the quality of life of patients with heart failure and educate them to perform individualized monitoring in order to detect worsening of their own symptoms, thereby avoiding rehospitalization. The FPT will be implemented and evaluated in a clinical trial. The aim of this paper is to describe the research design, outcome measures, and data collection techniques used in the Future Patient research project.

## Methods

### Future Patient Telerehabilitation Program

**Figure 1:**
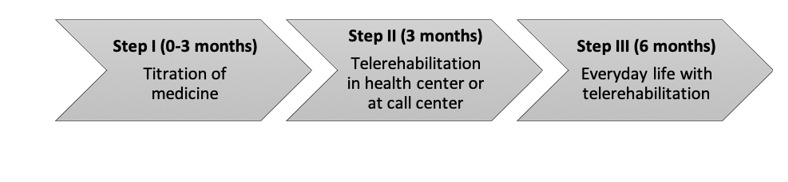
Telerehabilitation program in three steps.

The FPT consist of three phases: (1) titration of medicine (0-3 months), (2) participation in the FPT at a health care center or call center (3 months), and (3) follow-up with rehabilitation in everyday life (6 months). The three phases of the telerehabilitation program are illustrated in Figure 1.

### The HeartPortal

**Figure 2:**
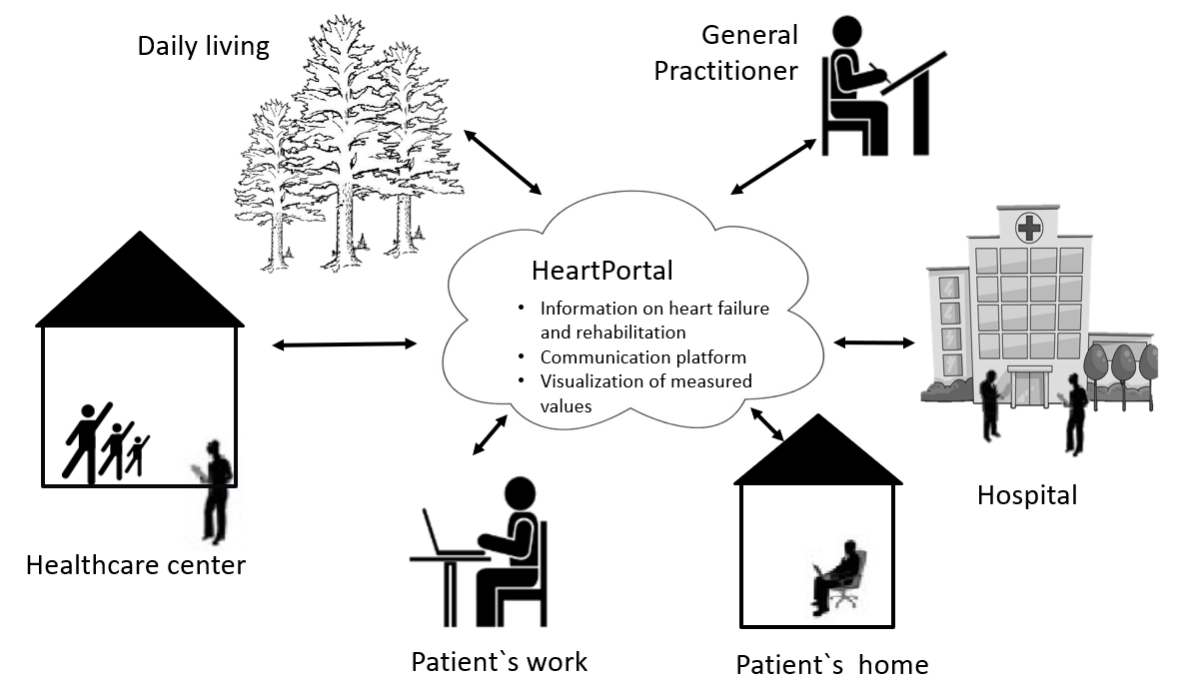
The context of the Future Patient study with the HeartPortal at the center.

**Figure 3:**
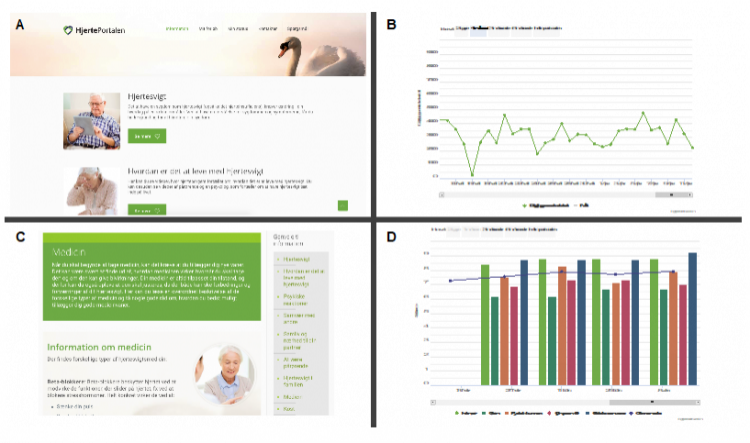
Screen captures from the HeartPortal. (A) Front page for the patients. (B) Measurement from the pedometer. (C) The information platform. (D) An illustration of the patient-reported outcome.

The HeartPortal is a digital toolbox that functions as an interactive learning module; the setup is illustrated in Figure 2, and screen captures are shown in Figure 3. The portal has been developed using a participatory design approach [19,21,22]. The HeartPortal consists of four elements:

- An interactive information site for patient education, containing information on rehabilitation issues, presented in the form of text and short videos (1-1.5 minutes) with patients and relatives describing their experiences with heart failure, symptoms, living an everyday life with heart failure, etc.
- A communication platform enabling patients to communicate directly with health care professionals. The HeartPortal may be used by health care professionals and patients to collaborate on setting goals and activities, and the patients also have the opportunity to keep an online diary and define their own goals for rehabilitation.
- Visualization of measured values (blood pressure, day and night pulse, weight, respiration, steps, and hours slept).
- Patient-reported outcomes (PRO) data [23]. Every second week, patients complete an online questionnaire about their sleep patterns (Spiegel Sleep Questionnaire) [24]; well-being (three questions defined by the research group); and measures on physical limitations, symptoms, self-efficacy, social interaction, and quality of life using the validated Kansas City Cardiomyopathy Questionnaire (KCCQ) [25,26]. The aim of the PRO-data is to give patients and health care professionals a digital tool to evaluate the patient’s current status. The data are presented in the tracking module, thus enabling both patients and health care professionals to view the PRO in all phases of the FPT.

As part of the telerehabilitation program, there is continuous monitoring of the patient’s vital signs using technology-enabled devices/equipment. A list of the equipment used is provided below:

- Blood pressure (UA 767PBT; A&D Medical, San Jose, CA) is measured every day during phase I.
- Weight scale (UC-321PBT; A&D Medical, Thebarton, South Australia, Australia) is used only in phase I of the FPT; in phases II and III, patients use their own weight scale. Patients with heart failure measure their weight every day during all three phases of the FPT.
- Data transmitter (QWH-HUB-V1.0E; Qualcomm Life, San Diego, CA) to help transfer data such as blood pressure, day pulse, and weight in a secure form from the devices and to the HeartPortal.
- Step counters (Fitbit Zip or Charge, San Francisco, CA). Patients with heart failure can decide what kind of stepcounter they want to use. Steps are measured every day and transmitted to the HeartPortal.
- Sleep sensor (Beddit 3, Espoo, Finland) measures the numbers of hours of sleep, night pulse, and respiration.
- A tablet (iPad Air 2, Cupertino, CA).

Figure 2 shows how data are presented in the HeartPortal. All the measured values are stored on a secure database at Aalborg University.

The patients receive instructions on evaluating their own values and how to react if these values are abnormal. A telerehabilitation coordinator observes all the measured values as well, and during phase 1, the coordinator also acts as a coach, helping patients learn to observe and act on any abnormal readings or worsening of symptoms to avoid possible hospitalization. During phase II, health care professionals also view the data. All health care professionals from the hospital and health care centers have access to the data.

### Data and Network Security

There is a strong focus on data and network security in the Future Patient project due to the project’s complexity and the environment where data are generated from both the health system (pulled data) and the patients (pushed data). Based on different endpoints in the setup (patient’s home, workplace, health care center, etc), the technology used, and the classification of information, the formation of metadata will be addressed via reliable integration encryption and infrastructure reliability.

### Eligibility Criteria

The target group in this study consists of patients diagnosed with heart failure according to the New York Heart Association (NYHA) class I-IV [27]. Patients with heart failure are recruited from the Cardiology Wards at hospitals in Skive, Viborg, Silkeborg, and Randers, all situated in the central region of Jutland, Denmark. The inclusion criteria are that the patient must have had a heart failure–related hospitalization within the past 2 weeks; have a stable internet connection at home; and have the ability to sign an informed consent form. Patients may have a pacemaker, and a maximum of 20% of the target group may belong to NYHA class I. Exclusion criteria are a revascularization or open-heart surgery within 3 months prior to inclusion; presence of previous neurologic, musculoskeletal, or cognitive disability or active psychiatric history (as noted in the medical record) other than depression or anxiety related to cardiac or other chronic illness; and absence of basic Danish language skills. All data from the recruitment process and patient withdrawal or dropout will be documented in a consort diagram.

### Research Design

The FPT will be tested in a multicenter randomized controlled trial (RCT) using a mixed method [28] approach. The intervention group will participate in the FPT, while the control group will follow a traditional rehabilitation program at the health care center, which is structured according to the national cardiac rehabilitation guidelines [29]. Enrollment of patients began in December 2016, and the RCT will end in September 2019.

### Power Calculation

The sample size was defined based on a power calculation. The aim of this project is to increase the quality of life (QoL) using telerehabilitation compared to usual rehabilitation. Based on the official KCCQ guidelines [25], the power calculation estimated the number of participants needed to show an increase in QoL on a scale of 0-100. When using the KCCQ, a “moderate” improvement is equal to a 10-point increase. The target group in the study includes patients with heart failure of a severe degree. As such, a 10-point improvement in the KCCQ will denote a change in the quality of life for the individual patient. Assuming normal distributions and a power of 80%, the number of participants in each group should be 63. However, accounting for 10% dropout, the total number of patients needed for this study is 140. Hence, we plan to enroll 70 patients with heart failure in the intervention group and 70 patients with heart failure in the control group.

### Theoretical Framework

The FTP program was designed using the self-determination theory (SDT) as a theoretical starting point. With the goal of increasing the effectiveness of behavior change interventions [30], SDT offers a framework for understanding the role of individualization in motivation. In particular, SDT highlights how fulfillment of basic needs such as autonomy, competency, and relatedness are necessary for initiating and maintaining changes in lifestyle and health behavior over time [31]. If motivation is sustained only by external factors, the patient will not have sufficient incentive to self-regulate and independently maintain a healthy lifestyle. However, if the patient finds that their health behavior goals are in accordance with their own internal values and beliefs (autonomy), the patient will feel that he/she possesses sufficient knowledge and skills to achieve this health behavior successfully (competency) and will feel supported by others (relatedness). The patient will, in turn, experience intrinsic motivation, that is, motivation based on internal factors enabling the patient to maintain the required lifestyle and health behavior over time [31].

### Ethics

The Future Patient project has been approved by the Regional Ethics Committee (N-20160055) and the Danish Data Protection Agency. The study is listed in listed in ClinicalTrials.gov (NCT03388918). The study is being carried out in accordance with the Helsinki Declaration, and all participants have signed an informed consent form prior to enrollment in the study.

### Baseline Data

Baseline data on demographics, clinical status, primary and secondary diagnosis, NYHA class, and actual prescribed medicines are being collected for both the intervention and control groups (Table 1). Questions on smoking, alcohol consumption, physical activity, IT competences, and rehabilitation will be asked in both groups in the RCT as well.

### Outcome Measures

Primary and secondary outcome measures as well as the dates when they the data will be collected are shown in Table 1. The data collection process is described below.

### Quality of Life

QoL is the primary outcome, which is measured using the KCCQ [25]. KCCQ is a 23-item self-administered questionnaire and includes different clinical domains such as physical limitations, symptoms, self-efficacy, social interaction, and quality of life. The score is calculated by assigning an ordinal value to each response, beginning with 1, and then adding up to make up a scaled score for each domain. Missing responses are assigned a value corresponding to an average of the answered items within the domain. Scale scores are transformed into a 0-100 range. In the intervention group, the QoL is measured at baseline and then every second week throughout the duration of the program. In the control group, QoL is measured at baseline, 6 months, and 12 months.

### Progression in Clinical data

All measured clinical data including weight, blood pressure, pulse (day/night), steps, sleep, and respiration in the intervention group will be collected and analyzed.

### Illness Perception

Changes in illness perceptions are being evaluated for both groups using the Brief Illness Perception Questionnaire (Brief IPQ) [32]. The Brief IPQ is a nine-item scale questionnaire designed to rapidly assess the cognitive and emotional representations of illness. Five of the items assess cognitive illness representation, two items assess emotional representation, and one item assesses illness comprehension. The last item is a causal question that asks the patient to list the three most causal factors in their illness. All items except one, the causal question, are scored on a scale from 0 to 10. The measure of the Brief IPQ is compared between groups at baseline, 6 months, and 12 months.

### Type of Motivation

Changes in the type of motivation are measured using the Health Climate Change Questionnaire (HCCQ) [33]. The HCCQ is a 15-item questionnaire that assesses patients’ perceptions of the degree to which their health care providers are supportive of their autonomy. Respondents rate each statement on a seven-point Likert scale (from “Not at all true” to “Very true”). The total score is calculated by averaging the values of each item. The HCCQ can be used in its full version (15-items) or short version (6-items). In this study, the full version is used. The scores from HCCQ are compared between groups at baseline, 6 months, and 12 months.

### Anxiety and Depression

Symptoms of anxiety and depression are measured using the Hospital Anxiety and Depression Scale (HADS) questionnaire [34]. HADS is a 14-item self-reported questionnaire consisting of two 7-item subscales measuring anxiety and depressive symptoms (ie, it does not assess somatic symptoms). Each item is scored on a four-point Likert Scale from 0 to 3. Scores are tabulated for each subscale, resulting in a score of 0-21, with higher scores indicating higher levels of anxiety or depressive symptoms. The measures from HADS are compared between groups at baseline, 6 months, and 12 months.

### Health and eHealth Literacy

Patients’ health and electronic health (eHealth) literacy skills are measured using a Danish validated version of the eHealth literacy assessment toolkit (eHLA) [35] and the eHealth Literacy Questionnaire (eHLQ) [36]. The eHLA is a toolkit used to assess health literacy and digital literacy. The toolkits consist of seven tools, four health related and three digitally related, that are either self-reported, such as questionnaires, or a performance test of relevant skills [35]. The eHLQ is a 35-item 7-scale questionnaire used to evaluate and understand how patients interact with digital health services [36]. The measurement of health and eHealth literacy skills is compared between groups at baseline, 6 months, and 12 months.

**Table 1:** Primary and secondary outcomes.

Outcome and measurement		Time of measurement					Group	
		Baseline	6 months	12 months	End of study	Continuous	Intervention	Control
**Primary**								
	Quality of life	✓	✓	✓			✓	✓
**Secondary**								
	Development of clinical data in intervention group			✓		✓	✓	
	Illness perception	✓	✓				✓	✓
	Type of motivation	✓	✓	✓			✓	✓
	Anxiety and depression	✓	✓	✓			✓	✓
	Health and eHealth^a^ literacy	✓	✓	✓			✓	✓
	Patients’ experiences		✓	✓			✓	
	Economic evaluation				✓		✓	✓

^a^eHealth: electronic health.

### Use of and Experiences Using the HeartPortal

Qualitative exploration of patients’, their relatives’, and health care professionals’ experiences using the HeartPortal will be conducted. Semistructured interviews inspired by Brinkman and Kvale [37] will be conducted after 6 months and 12 months. To analyze which parts of the HeartPortal are being used and for how long, time log files for login/logout of patients and relatives will be analyzed. The patients and relatives will be asked for their consent to extract their log files from the database for analysis.

### Economic Evaluations

A cost-effectiveness analysis will be based on the guidelines for economic evaluation by Drummond et al [38]. Estimates of the mean costs per patient will be made with a broad societal perspective, including use of resources for patients, hospital, and municipality. Estimation of costs will be based on data at the patient level for all patients, both at the intervention and control groups.

### Data Analysis

All demographic data are being stored in the Future Patient database (FPD). Data from questionnaires are stored in Research Electronic Data Capture software [computer software] (Nashville, TN: Vanderbilt University) and used for quantitative analysis.

### Adverse Events and Dropout

All adverse events, including deaths, dropouts, or withdrawals from the study will be recorded and documented. If the patients no longer want to participate in the study, they can withdraw their consent at any time, and the reason for withdrawal will be documented. In this case, the project team will collect the equipment upon request. Patients who do not participate actively in the intervention will still be included in the study and analyzed according to the intention-to-treat approach. These patients will be allowed to retain use of the project equipment for as long as they want. Technical problems with the equipment will be recorded and documented.

### Statistical Analysis

At baseline, descriptive statistics [39] will be reported as median with 25th-75th percentiles in the case of skewed distribution or mean (SD) for normally distributed continuous variables. The nonparametric Kolmogorov-Smirnov test will be used to investigate normality of the distribution. An intention-to-treat analysis on all the randomized subjects will be conducted to provide unbiased comparisons between the intervention and control groups in order to avoid the effects of dropout.

The analysis of change in QoL, anxiety and depression, self-determination, illness perception, type of motivation, and health and eHealth literacy tests will be performed using data acquired through questionnaires. Comparison of incidence rates will be used to investigate the differences between the intervention and control groups. Two-sided tests and a significance level of 0.05 will be used.

All events from the day after randomization to patient exit will be included, while any other evident outcomes will be measured as changes from baseline to all assessment time points. Changes in the other evident outcomes will be tested using linear mixed models. Linear mixed models allow repeated measures to be collected in a longitudinal design and are superior when dealing with dropouts compared to other methods used for repeated measures. Hence, it will not be necessary to use imputation techniques on missing data. Statistical analyses will be performed using SPSS [computer software] (version 25.0. Armonk, NJ: IBM Corp), and values of *P*<.05 will be considered significant for all tests.

### Qualitative Analysis

All interviews will be transcribed into text files by a research assistant. The data will be coded in NVivo [computer software] (version 12.0. Melbourne, Victoria, Australia: QSR International; 2018), with inspiration from the SDT theoretical framework and based on methods developed by Brinkman and Kvale [37]. Two researchers will conduct the interviews and analysis of the data. The date will be presented in themes and findings.

## Results

Results from the RCT will be analyzed in the fall of 2019 and be published in peer-reviewed journals in the fields of telerehabilitation, clinical cardiology, and health economics and be presented at international conferences.

The evaluation of the Future Patient Telerehabilitation program includes the clinical data recorded by the patient and psychosocial, health literacy, and eHealth literacy through questionnaires. Qualitative exploration of the perspectives of patients with heart failure, relatives, and health care professionals as well as a health economic evaluation will be conducted. The results from the RCT will be analyzed, and the results are expected to be published by spring 2020.

## Discussion

### Telerehabilitation Program

The aim of the Future Patient study is to test, implement, and evaluate a telerehabilitation program for patients with heart failure. The RCT was divided into three phases in the intervention group: a posthospitalization phase, a rehabilitation phase, and daily life adjustment 6 months after rehabilitation. A more holistic approach to patient rehabilitation is introduced on the basis of user-driven innovation, to accommodate the needs of the end user. This was just the first step toward engaging the patient in self-management. The uniqueness of this study lies in two areas: the use of a variety of self-monitoring methods and the use of SDT within a telerehabilitation context. The measurements include activity, pulse, and sleep trackers, which automatically transmit the monitored data to the HeartPortal. The HeartPortal has the potential to facilitate and educate patients in self-managing their disease. In this way, patients can become more engaged in their disease progression. The patient has the option of sharing their data, thus providing the patient’s relatives the opportunity to monitor the progress of the patient through the HeartPortal, which, in turn, enables the relatives to motivate and support the patient according to their progression. The PRO data is a new tool that we will be tested, and we have not identified other studies that are working with PRO data for patients with heart failure.

The expected benefit of the health economy evaluation for the telerehabilitation group compared with the control group is increased quality of life, reduced contacts to outpatient clinics at the hospital, and a reduction in admissions to hospitals.

The HeartPortal is also designed to enable the patients to improve their eHealth literacy through a combined information platform. In addition, a communication platform has been incorporated into the HeartPortal, from which the patient and his/her relative can communicate with health care professionals, psychologists, and technical personnel. As such, the relative is an important member in this study, as studies have found that support from family and friends benefits patients with heart failure by improving patients’ quality of life and reducing rehospitalizations [40]. Additionally, support from family and friends may promote self-management behaviors in the patient with heart failure, facilitating increased medication and dietary adherence [40].

### Limitations

This study had three limitations that should be considered. First, a subject group consisting of patients with heart failure is highly sensitive to death, as this group is dominated by the elderly [40]. Second, the intervention period extends to 1 year and is therefore highly dependent on the sustained motivation of the subjects. Third, as the tracking devices used in the study are commercial products purchased for the purpose of automatically transmitting data to the HeartPortal, the study is highly dependent on the data transmission and access hereof. If the regulations for the devices change during the study period, it would be necessary to replace them with other devices, which may lead to inconsistency in biases.

### Conclusions

The expected outcomes are increased quality of life, increased motivation and illness perception, reduced anxiety and depressions, improved eHealth literacy, and health economics benefits. We expect the study to have a clinical impact for future telerehabilitation of patients with heart failure.

## Abbreviations

eHealth: electronic health
eHLA: eHealth literacy assessment toolkit
eHLQ: and the eHealth Literacy Questionnaire
FPD: Future Patient database
HADS: Hospital Anxiety and Depression Scale
HCCQ: Health Climate Change Questionnaire
IPQ: Illness Perception Questionnaire
KCCQ: Kansas City Cardiomyopathy Questionnaire
NYHA: New York Heart Association
PRO: patient-reported outcomes
QoL: quality of life
RCT: randomized controlled trial
SDT: self-determination theory
